# The Association between Gene-Environment Interactions and Diseases Involving the Human GST Superfamily with SNP Variants

**DOI:** 10.3390/ijerph13040379

**Published:** 2016-03-29

**Authors:** Antoinesha L. Hollman, Paul B. Tchounwou, Hung-Chung Huang

**Affiliations:** 1 NIH/NIMHD RCMI Center for Environmental Heath, College of Science, Engineering, and Technology (CSET), Jackson State University, Jackson, MS 39217, USA; paul.b.tchounwou@jsums.edu; 2 Department of Biology, CSET, Jackson State University, Jackson, MS 39217, USA

**Keywords:** gene-environment interactions, glutathione S-transferases (GSTs), single nucleotide polymorphisms (SNPs), carcinogens, xenobiotics, heavy-metals, air pollutants

## Abstract

Exposure to environmental hazards has been associated with diseases in humans. The identification of single nucleotide polymorphisms (SNPs) in human populations exposed to different environmental hazards, is vital for detecting the genetic risks of some important human diseases. Several studies in this field have been conducted on glutathione S-transferases (GSTs), a phase II detoxification superfamily, to investigate its role in the occurrence of diseases. Human GSTs consist of cytosolic and microsomal superfamilies that are further divided into subfamilies. Based on scientific search engines and a review of the literature, we have found a large amount of published articles on human GST super- and subfamilies that have greatly assisted in our efforts to examine their role in health and disease. Because of its polymorphic variations in relation to environmental hazards such as air pollutants, cigarette smoke, pesticides, heavy metals, carcinogens, pharmaceutical drugs, and xenobiotics, GST is considered as a significant biomarker. This review examines the studies on gene-environment interactions related to various diseases with respect to single nucleotide polymorphisms (SNPs) found in the GST superfamily. Overall, it can be concluded that interactions between GST genes and environmental factors play an important role in human diseases.

## 1. Introduction

Human health or disease development is highly influenced by interactions between gene expression and environment [1]. Glutathione S-transferase (GST) is a dimeric, multifunctional protein superfamily, present in all kingdoms [2,3]; which has sparked an interest in the area of gene-environment interaction and diseases. Recent findings have demonstrated the importance of different allelic frequencies of polymorphic genes, such as GSTs, and susceptibility to certain diseases [4]. Changes or variations in an individual’s DNA, such as single nucleotide polymorphisms (SNPs), are deemed to produce numerous diseases [5,6].

GSTs are vital in Phase II detoxification enzymes pathway in humans, and provide protection against toxins by catalyzing toxin conjugation with GSH or passively binding to various exogenous/endogenous toxic molecules, including environmental toxins, carcinogens, chemotherapeutic agents, or products of oxidative stress [3,7]. They are also involved in preventing cellular mutations and aiding in antioxidant defense mechanism. However, there are some instances where conjugation reactions can lead to the formation of compounds that are far more toxic than the initial substrate, thereby potentially causing a disease [8]. Some GSTs, which undergo polymorphisms, pose an interest in exploring the relationship between specific allelic variants and the risk of developing a disease [9,10,11,12,13]. Therefore, our goal is to examine studies on gene-environment interactions related diseases, with respect to SNPs found in the GST subfamilies as shown in Table 1 [14].

The mechanisms for the genetic variances can be complex. Conventionally there are two kinds of mutations, germinal and somatic mutations [15,16]. Germinal mutations can be passed down to offspring generations to become inherited mutations or polymorphisms. Inherited polymorphisms, unlike somatic mutations, are congenital and not induced by environmental factors. Somatic mutations develop at some stage of the cell lifespan due to exposure to environmental hazards. The focus of this article is on the SNP polymorphisms caused by somatic mutations induced by environmental risk factors. Nonetheless, the subtle differences (variances) of inherited genetics for different race/ethnic group populations in different locations, may confer different susceptibilities to somatic mutations induced by some factors (Table 2).

## 2. Glutathione S-transferases (GSTs)

The first GST structure, in 1991, leads to an overflow of structural data among GSTs of the three distinct superfamilies: the cytosolic GSTs (largest), the mitochondrial GSTs and microsomal, also known as the membrane-associated proteins in eicosanoid and glutathione metabolism (MAPEG family) [17]. Several mammalian GST classes have been identified and characterized, forming eight distinct classes: alpha, mu, pi, omega, sigma, theta, zeta, and kappa; with the first seven being cytosolic specific [18,19]. Alpha, mu, and pi are noted being the major classes [20] with their SNP distributions shown in Table 1 [14]. An extensive amount of literature has been published on theta, as well [14,21], which SNP distribution can also be seen in Table 1. In some cases, GSTM1 (glutathione S-transferase mu), GSTT1 (glutathione S-transferase theta), and GSTP1 (glutathione S-transferase pi) are evaluated in experiments altogether. GSTs are present in virtually all tissues, yet in humans the liver has the highest cytosolic GST activity level. Those major cellular detoxification enzymes are present mostly in the liver and kidney, as well as in the intestine [22].

The four widely studied GSTs (GSTA, GSTM, GSTP, and GSTT) are parts of the cytosolic GST group and are involved in the detoxification of xenobiotics, carcinogens, and therapeutic drugs. Hence, they are deemed insightful biomarkers as some of their polymorphic states can increase an individual or population’s susceptibility to a disease (e.g., cancer). Class alpha GST (GSTA), located on chromosome 6 and highly expressed in the liver, plays a role in cell protection in the presence of peroxidation products and reactive oxygen species (ROS). It can serve as an indicator for liver injury, as it can be detected at lower levels in acute hepatic injury [23]. Class mu (GSTM), located on chromosome 1, is known to modify the toxicity and effectiveness of medical drugs. Class pi (GSTP), located on chromosome 11, is involved in the protection of cells from cytotoxic and carcinogenic agents [24]. Lastly, class theta (GSTT), located on chromosome 22, shares a similarity with 55% amino acid sequence identity and possesses a possible role in human carcinogenesis. Most GSTs’ functions are associated with detoxification or anti-oxidation processes. Therefore, their structural changes due to SNP variants (especially missense mutations) have a strong correlation with chronic diseases such as cancers.

Glutathione S-transferase alpha (GSTA) family consists of GSTA1, GSTA2, GSTA3, GSTA4, GSTA5, GSTA6P (pseudogene), and GSTA7P (pseudogene) that are located on chromosome 6. This class encodes enzymes with glutathione peroxidase activities that function in the detoxification of lipid peroxidation products. Glutathione S-transferase mu (GSTM) family includes GSTM1, GSTM2 (muscle), GSTM3 (brain), GSTM4, and GSTM5, found on chromosome 1. GSTM1 enzyme encodes a major detoxification phase enzyme that helps detoxify various xenobiotics. Deficiency in GSTM1 activity is related to homozygous deletion of GSTM1 (GSTM1 null), leading to a lack of corresponding enzymatic activity [8,25]. Glutathione S-transferase pi (GSTP1) has an enzymatic activity that provides a “caretaker” function. It has been documented that inactivation of the GSTP1 gene was often observed in human neoplasia (prostate, breast, and liver cancer, as well as leukemia) and researchers highlight GSTP1 epigenetic modifications as biomarkers for early diagnosis for cancers and potential targets of preventive or therapeutic treatments [26,27]. Glutathione S-transferase theta (GSTT) family is comprised of GSTT1, GSTT2 (gene/pseudogene), and GSTT2B (gene/pseudogene) and is positioned on chromosome 22. This gene is polymorphic in human and the null genotype results in the absence of enzyme function, which may influence alterations in the response of xenobiotics. In recent years, many studies have assessed the associations between diabetes mellitus (DM), Type 2 diabetes mellitus (T2DM) and GSTT1 polymorphism; although no significant association was found [28], GST polymorphic genes (GSTM1-null and GSTT1-null) can be used as biological markers to determine the diabetic risk of individuals [4].

DNA mutations are associated with many human diseases and are the reasons for the variations among individuals. Many polymorphisms in the DNA sequence of these GSTs are reported and many studies have demonstrated that the polymorphisms of these GSTs are associated with different types of cancer [5,6,29,30]. As studied by Yadav *et al.* [14] based on NCBI/dbSNP database, the major studied GST genes [14] contained 3193 SNPs with 237 coding nsSNP among them (Table 1) which should be the focus of the investigation due to their potential effects on the structure, function, interactions, and other properties of DNA and expressed proteins. Obviously, intron noncoding SNPs consist of the most percentage (84.87%) of total SNPs under study; these intronic SNPs will not be the focus of our current review, although some of them may relate to regulatory or splicing mechanism. Significant coding nsSNPs will be identified and highlighted in the next section.

## 3. GST Single Nucleotide Polymorphisms (SNPs)

GST genes are organized in chromosomal clusters, and most of these genes are polymorphic, mainly due to single nucleotide substitutions or variations (*i.e.*, SNPs). Genetic variations can be classified as synonymous or nonsynonymous. Synonymous variation is the alteration in the DNA, which produces a change in the amino acid due to the nucleotide change, but does not affect the function. However, missense, nonsense, and frameshift changes are types of nonsynonymous mutations that all generate a significant change in the protein. In recent years, a few researchers suggested that deleterious nonsynonymous single nucleotide polymorphisms (nsSNP) of GSTs are associated with diseases. One study [14] discovered that five (GSTA3/R13W, GSTA3/Y147D, GSTM3/R191L, GSTM4/R18L, and GSTT1/W101R) of 237 nsSNP were identified as potential target mutations that induced structural changes and possibly alter the detoxification process that could lead to carcinogenesis events (Table 3). 

It has also been recognized that there are modifications in enzymatic activity of a missense categorized SNP in GSTP1 (rs1695/Ile105Val) at residue 105 that leads to miscellaneous diseases [9,10,11,12,13]. Internationally, it has been determined that the GSTP1 Val105 allele occurs more frequently in African-Americans (42%) and European-Americans (33%) *versus* other ethnicities, such as Chinese (22%), Taiwanese (18%), and Japanese (14%) populations, while in the nsSNP Ala114Val, GSTP1 Val114 allele is not common in African-Americans (5%) or European-Americans (9%) [41].

### 3.1. Significant GST SNPs

Based on the literature reviewed, we compiled the most significant GST SNPs (related to disease) in Table 3. The criteria used to obtain information for this paper were based on the major GST-related & disease-associated SNPs found in PubMed, a service that provides access to literature, and dbSNP, NCBI’s database containing genetic variation data. In Table 3, we included the significant or potential SNPs that fit our criteria which are: (1) the SNP was located in GST gene or close to it; (2) the SNP was studied and described in some literature(s) in PubMed; (3) the SNP has a dbSNP ID; (4) the SNP formation was susceptible to some kind of environmental factor including variances in ethnic group and living locations; (5) the SNP was associated with a disease or has the potential to cause a disease. Although some significant SNPs are located in the noncoding areas (e.g., GSTM5/rs3754446, GSTA1/rs3957357, GSTP1/rs4147581, GSTP1/rs947895, GSTT1/rs4630, GSTO2/rs7085725, and GSTZ1/rs1468951 in Table 3), most significant GST SNPs are found in the coding areas which can have a strong effect on the structure and function of the translated protein due to consequent amino acid change on the SNP site. Most of the SNPs in Table 3 have a connection with the environmental factors including carcinogen, toxin, heavy metal, cigarette smoke, air pollutant, UV exposure, and other environmental hazards. GSTA2 (P110S, S112T, and E210A) are in linkage disequilibrium (with one another), as shown in Table 3, which displays potential damage by these three GSTA2 variants collectively [42] although it has only been shown that GSTA2 S112T serine allele homozygosity is a prognostic factor for poorer survival, for increased any time- and 100-day transplant-related mortality [43]. It was determined that some contributing factors related to SNP formation can be associated with ethnic group’s different susceptibility, location for industrial waste toxin or heavy metal, and other environmental factors. Our focus is on the somatic variances that are associated with environmental factors which may induce a potential disease, including the possibilities of disease susceptibility depending on a specific ethnic/population group with different living habits in a certain location (Table 2). In addition, most GST SNPs are linked to carcinogenesis.

### 3.2. Databases for GST SNP Studies

Based on the literatures published so far, we found that dbSNP, HapMap, and HGDP datasets are often utilized and examined for the GST SNP analyses (Table 4). Also, SNPedia and PharmGKB are useful for SNP annotations (Table 4). There are additional SNP datasets to be explored in more recent projects as seen in the databases of COSMIC, ICGC, and TCGA which focus on the genetic changes in cancers (Table 4).

### 3.3. Programs for GST SNP Analyses

Based on the reviews, there are many programs for SNP analyses and depending on the stage of the analyses, specific program should be used in each stage. For simplicity, we selected a few typical and popular programs to be included in Table 5 for an overall view of the whole process in SNP data analyses. For example, GATK [76] can be the first program to manage the high-throughput raw data for SNP variant discovery and/or genotyping, PLINK [77] can be used to do genome wide association study (GWAS), VEGAS (VEGAS2) [78,79] prepares a gene-based association test via SNP GWAS results, Arlequin [69,80] can perform population allele comparison and analysis of molecular variance (AMOVA), Haploview [69,81] is capable of producing visualizations and plots for the PLINK GWAS results, R/QTL [82] is able to generate a quantitative trait loci analyses to pinpoint the causative SNP loci for the disease, and finally, Triton, SIFT, and Polyphen2 [14,83,84,85,86] can evaluate and predict if the SNP is damaging or not (Table 5). Starting from these programs, some more similar or related programs can be identified.

## 4. Environmental Hazards and Diseases Associated with GST Gene-Environment Interaction

Living organisms encounter exposure to various toxins and/or toxicants, *i.e.*, industrial chemicals, pesticides, herbicides, air pollutants, pharmaceuticals and several natural occurring substances, that can have a detrimental effect on a human’s health. These environmental exposures can induce changes in gene regulation associated with human diseases [7]. Exposure to the same environment does not warrant the same effect on different individuals within or outside of a particular ethnicity, due to the differences in a person’s DNA. For example, genome-wide association studies (GWAS) have identified a number of genetic variants connected with the risk of bladder cancer in populations of European descent [87].

A correlation among gene polymorphisms and environmental toxins, such as heavy metals (arsenic, lead, and platinum), air pollutants, and other factors as seen in Table 6, have been the focus for some studies to display the potential risks [88]. Some findings provide information on exposures to environmental lead and an analysis of blood lead levels in men that exhibited genetic polymorphisms in GSTs (deletions of GSTM1/GSTT1 and GSTP1 rs1695), resulting in adverse alterations in inflammatory response [9]. Also, mothers exposed to environmental tobacco smoke show an increased probability of a negative impact on birth weight (*i.e.*, low birth weight) [25]. There is an association between cancer incidence and various disorders of GSH-related enzyme functions especially the alterations of glutathione S-transferases (GSTs) [89]. It has been suggested that GSTM1/T1 polymorphisms are related with many diseases, such as rheumatoid arthritis, age-related macular degeneration, oral leukoplakia, prostate cancer, lung cancer, and cervical neoplasia [14,45,52,53,90]. GSTM1 null genotype and GSTP1 Ile105Val polymorphism are associated with the increased risk of Alzheimer's disease [12]. The pi class GST (P1) is often overexpressed in human tumors, including carcinomas of the colon, breast, lung, kidney, ovary, pancreas, esophagus, stomach, prostate, liver, and blood [19,27]. In relation to prostate cancer, there are no consistent associations between GSTM1, GSTT1 or GSTP1 genotypes [91] and related studies produced as recent as 2012 give the same information. Although one study states that its findings revealed no apparent interaction between GST gene variants and hypertension due to exposure to air pollution [21], there are instances where polymorphisms of GSTs are involved (positively or negatively). GSTO1 (glutathione S-transferase omega) related SNP in arsenic (As) metabolism exhibited nominally significant interactions with well-water “As” for connections with cardiovascular disease (CVD), coronary heart disease (CHD), or stroke [92]; in addition, GSTT1 polymorphisms serve as a potential genetic factor for arsenic-induced skin cancer [93]. It is suggested that GSTP1 aids in the detoxification of arsenic [13]. The GSTT1 also encodes enzymes involved in the metabolism and detoxification of polycyclic aromatic hydrocarbons (PAHs), and the protection against genotoxic damage due to the ethylene oxide present in tobacco smoke [25].

In Table 6, it is illustrated that the diseases related to GST SNP variants can be classified into five categories and most of the affecting factors have an association with the environmental toxins or toxicants. The criteria for the creation of this table are to focus on classification of the diseases highly related to SNPs found in GSTs. These five categories of disease are: (1) Cancers; (2) Inflammatory or Immunological Disorders; (3) Neurological Disorders; (4) Aging-related or Metabolic Disorders; and (5) Reproductive Disorders. Environmental toxins are the most important affecting factors for all five categories of diseases and are the causing factors for all cancers, Inflammatory or Immunological Disorders, and Reproductive Disorders.

## 5. Conclusions

The findings reviewed in this article display the role of environmental factors and how they influence the genome and its regulation, providing the clue that xenobiotics found in the environment as a result of anthropogenic activities can promote disease by altering gene allele. The study of gene-environment interactions is relevant in improving the human health, as researchers seek to determine risk factors that are potentially due to environmental exposures that produce differences in gene sequences [97]. This would aid in understanding and determining the initiation of the disease and would enhance the chance of protection against those diseases. Though GSTs’ detoxifying activity aids in the protection of cells from certain diseases, they are also vulnerable to environmental toxin or hazard for the gene allele change leading to some life-threatening diseases. Hence, we postulate that interactions between GST genes and environmental factors play an important role in adverse health effects among humans. In addition, from recent international projects, there are more cancer SNP datasets available that haven’t been fully explored yet, and their detailed examination and analyses in the future can identify more GST SNPs related to various cancers. This can be one step further to approach the practice of gene therapy (editing) via CRISPR/Cas9 [98,99,100] or disease treatment via personalized precision medicine [101] in the future.

## Figures and Tables

**Table 1:** Major types of GST genes and their SNP distributions (based on [14]).

Gene Family	Genome Location	Total SNP	nsSNP	% nsSNP	sSNP	% sSNP	3′UTR	% 3′UTR	5′UTR	% 5′UTR	iSNP	% iSNP
GSTM	Chr1	1072	92	8.58	53	4.94	55	5.13	8	0.75	864	80.60
GSTA	Chr6	1702	98	5.76	43	2.53	34	2.00	21	1.23	1506	88.48
GSTP	Chr11	180	17	9.44	6	3.33	3	1.67	6	3.33	148	82.22
GSTT	Chr22	239	30	12.55	11	4.60	3	1.26	5	2.09	192	80.33
Total		3193	237	7.42	113	3.54	95	2.98	40	1.25	2710	84.87

nsSNP: non-synonymous SNP; sSNP: synonymous SNP; 3′UTR: 3′ untranslated region; 5′UTR: 5′ untranslated region; iSNP: intronic SNP.

**Table 2:** Population susceptibility for different diseases related to major types of GST.

Gene Family	Population Susceptibility
GSTM	Chinese (LAC) [31], Indian (OC) [32], North Indian (CC) [33], Caucasians (CRC) [34], Iranian (BC) [35]
GSTA	Brazilian (PCa) [36]
GSTP	Asian (BC) [37], Iranian (BC) [35], Saudi Arabian (DLBCL) [38]
GSTT	North Indian (CC) [33], Chinese (LC) [39], Caucasians (CRC) [34], Chinese (GC) [40], Saudi Arabian (DLBCL) [38]

Abbreviations: BC—Breast Cancer; CC—Cervical Cancer; CRC—Colorectal Cancer; DLBCL—Diffuse Large B-cell Lymphoma; GC—Gastric Cancer; LAC—Lung adenocarcinoma; LC—Lung Cancer; OC—Oral Cancer; PCa—Prostate Cancer.

**Table 3:** Significant GST SNPs related to miscellaneous diseases or mechanisms.

Variation	dbSNP ID	SNP Category	Nucleotide Change	Affecting Factor	Related Disease or Mechanism	Reference
GSTA1/NG5-69C/T	rs3957357	Noncoding	C–T	Ethnic Group, Location	Asthma, Allergy	[44]
GSTA2/P110S	rs2234951	Missense	C–T	Environment	Strong LD *	[42]
GSTA2/S112T	rs2180314	Missense	G–C	Environment	Transplant-related mortality, Strong LD *	[42,43]
GSTA2/E210A	rs6577	Missense	A–C	Environment	Strong LD *	[42]
GSTA3/R13W	rs59410661	Missense	A–G	Universal	Carcinogenesis	[14]
GSTA3/Y147D	rs144126679	Missense	A–C	Universal	Carcinogenesis	[14]
GSTM1/K173N	rs1065411	Missense	G–C	Cigarette Smoking	Colorectal Cancer	[45]
GSTM1/T154S	rs135955605	Missense	C–G	Climate, Environment	Bull Sperm Quality	[46]
GSTM3/R191L	rs1803686	Missense	C–A	Universal	Carcinogenesis	[14]
GSTM4/R18L	rs138088784	Missense	G–T	Universal	Carcinogenesis	[14]
GSTM5/NG5	rs3754446	Noncoding	T–G	Ethnic Group	AML	[47]
GSTP1/I105V	rs1695	Missense	A–G	Arsenic, Carcinogenic Compounds	Asthma, BC, Inflammation, Gastric Cancer, Autism and Alzheimer’s	[9,10,11,12,13]
GSTP1/A114V	rs1138272	Missense	C–T	Ethnic Group	MND	[41,48]
GSTP1/S185S	rs4891	Synonymous	T–C	Cigarette Smoking	Lung Cancer	[49]
GSTP1/intron	rs4147581	Noncoding	G–C	Ethnic Grp., Location	HCC	[50]
GSTP1/NG3	rs947895	Noncoding	C–A	Ethnic Grp., Location	Asthma	[51]
GSTT1/V51I/V169I	rs2266637	Missense	G–A	Ethnic Group	Carcinogenesis	[52,53]
GSTT1/W101R	rs141759372	Missense	A–G	Universal	Carcinogenesis	[14]
GSTT1/3′-UTR	rs4630	Noncoding	C–T	Thalidomide	Peripheral Neuropathy	[54]
GSTO1/A112D/A140D	rs4925	Missense	C–A	Ethnic Grp., Location	Alzheimer Disease	[55]
GSTO2/N142D	rs156697	Missense	A–G	Smoking, UV exposure	Cataract, Asthma, Lung function	[44,56,57]
GSTO2/3′-UTR	rs7085725	Noncoding	T–C	Ethnic Grp., Location	HCC	[50]
GSTZ1/E32K	rs7975	Missense	G–A	Cigarette Smoking	Carcinogenesis	[58,59]
GSTZ1/intron	rs1468951	Noncoding	C–A	Ethnic Group	Cognition	[60]

Abbreviations: BC, Breast Cancer; LD, Linkage Disequilibrium; AD, Alzheimer’s Disease; NG5, Near-Gene-5; AML, Acute myeloid leukemia; NG3, Near-Gene-3; MND, Motor Neuron Disease; HCC, Hepatocellular Carcinoma; Missense, Missense SNP; Synonymous, Synonymous SNP; UTR, UnTranslated Region. (Note 1: Universal factor can be environmental or genetic; Note 2: NG5 (Near-gene-5), near 5′ end of the gene; Note 3: NG3 (Near-gene-3), near 3′ end of the gene.).* Strong LD: GSTA2 (P110S, S112T, and E210A) are in linkage disequilibrium (with one another), which displays potential damage by these three GSTA2 variants collectively [42] although it has only been shown that GSTA2 S112T serine allele homozygosity is a prognostic factor for poorer survival, for increased any time- and 100-day transplant-related mortality [43].

**Table 4:** Databases for SNP studies.

Database	Focus	Link	Reference
1000 Genomes	Human genetic variation	http://www.1000genomes.org	[61]
COSMIC cancer database	Somatic mutations in cancer	http://cancer.sanger.ac.uk/cosmic	[62]
dbGAP	Genotype, Phenotype	http://www.ncbi.nlm.nih.gov/gap	[63,64,65]
dbSNP	SNP	http://www.ncbi.nlm.nih.gov/SNP	[14,63,64]
HapMap	Haplotype map of human genome	http://hapmap.ncbi.nlm.nih.gov	[66,67,68,69,70]
HGDP	Genetic diversity in humans	http://www.hagsc.org/hgdp	[69,70,71]
ICGC	Oncogenic mutations	https://icgc.org	[72]
PharmGKB	Genetic variation on drug response	https://www.pharmgkb.org	[52,73]
SNPedia	Effect of DNA variations	http://www.snpedia.com/index.php/SNPedia	[74]
TCGA	Genetic changes in cancers	http://cancergenome.nih.gov	[75]

**Table 5:** Programs for SNP analyses.

Program	Application	Link	Reference
GATK	Variant discovery and genotyping	https://www.broadinstitute.org/gatk	[76]
PLINK	Genome association analysis toolset	http://pngu.mgh.harvard.edu/~purcell/plink	[77]
VEGAS and VEGAS2	Gene-based test for association via SNP association results	http://gump.qimr.edu.au/VEGAS; https://vegas2.qimrberghofer.edu.au	[78,79]
Arlequin	Population allele comparison and AMOVA	http://cmpg.unibe.ch/software/arlequin35	[69,80]
Haploview	Haplotype analysis	https://www.broadinstitute.org/scientific-community/science/programs/medical-and-population-genetics/haploview/haploview	[69,81]
R/QTL	Mapping quantitative trait loci	http://www.rqtl.org	[82]
Triton	Protein mutant construction and activity prediction	http://www.ncbr.muni.cz/triton	[14,83,84]
SIFT	Predict amino acid substitution effects	http://sift.bii.a-star.edu.sg/	[14,85]
Polyphen2	Predict damaging missense mutation	http://genetics.bwh.harvard.edu/pph2	[14,86]

**Table 6:** Miscellaneous diseases or mechanisms related to GST variants.

Disease or Mechanism	Affecting Factor	Related GST	Reference
**Cancers**			
Blood Cancer	Misc. Carcinogen and Toxin	GSTM5, GSTP1	[27,47]
Breast cancer	Misc. Carcinogen and Toxin	GSTP1	[27]
Colorectal cancer	Misc. Carcinogen and Toxin	GSTM1	[45]
Gastric cancer	Misc. Carcinogen and Toxin	GSTP1	[11]
Liver cancer	Misc. Carcinogen and Toxin	GSTP1, GSTO2	[27,50]
Lung problem or cancer	Air pollutant, Smoke, Platinum, Carcinogen	GSTM4, GSTP1, GSTO2, GSTZ1	[49,57,59,94]
Prostate cancer	Misc. Carcinogen and Toxin	GSTP1	[27]
Skin cancer	Arsenic	GST M1, P1, T1, and O1	[92,93]
**Inflammatory or Immunological Disorders**			
Asthma	Air pollutant, Smoking, and Toxin	GSTA1, GSTP1, GSTO2	[9,10,11,44,51]
Allergy	Air pollutant, Carcinogen, Toxin	GSTA1	[44]
Inflammation	Lead (Pb)-induced	GSTM1, GSTP1, GSTT1	[9]
**Neurological Disorders**			
Alzheimer’s disease	Genetics	GSTM1, GSTM3, GSTP1, GSTO1	[12,55,95]
Autism spectrum disorder	Arsenic	GSTP1	[13]
Brain cognition	Environment	GSTZ1	[60]
Motor neuron disease	Genetics	GSTP1	[48]
Peripheral neuropathy	Immunomodulatory drug	GSTT1	[54]
**Aging-related or Metabolic Disorders**			
Age-related cataract	Smoking, UV exposure	GSTM1, GSTT1, GSTO1, and GSTO2	[56,90]
Cardiovascular disease	Arsenic	GSTM1, GSTT1, GSTO1	[92]
Hypertension	Air pollutant	GSTP1	[21]
Type 2 diabetes	Weight, diet, race, and genetics	GSTM1,GSTT1	[4]
**Reproductive Disorders**			
Fetal growth restriction or adverse pregnancy outcome	Environment, smoking, pesticide	GSTM1, GSTT1	[25,88,96]
Spermatogenesis	Species, environment	GSTM1	[46]
